# Mapping Potential Distribution of *Spodoptera frugiperda* (Lepidoptera: Noctuidae) in Central Asia

**DOI:** 10.3390/insects11030172

**Published:** 2020-03-09

**Authors:** Muhammad N. Baloch, Jingyu Fan, Muhammad Haseeb, Runzhi Zhang

**Affiliations:** 1 Institute of Zoology, Chinese Academy of Sciences, Beijing 100101, China; naveedmalkani@ioz.ac.cn (M.N.B.);; 2 University of Chinese Academy of Sciences, Beijing 100049, China; 3 Center for Biological Control, College of Agriculture and Food Sciences, Florida Agricultural and Mechanical University, Tallahassee, FL 32307, USA; muhammad.haseeb@famu.edu

**Keywords:** biological invasion, maize, pest, ecological niche model, risk analysis

## Abstract

*Spodoptera frugiperda* is a serious agricultural pest native to tropical and subtropical areas of the Americas. It has a broad host suitability range, disperses rapidly, and has now invaded nearly 100 countries around the world by quickly establishing in the novel ecologies. Based on the native occurrence records and environmental variables, we predicted the potential geographic distribution of *S. frugiperda* in Central Asia using the MaxEnt model and the ArcGIS. Irrigation is considered to be the main factor for the maize crop production in the Central Asia; therefore, we sought to map the potential spread of *S. frugiperda* using two modeling approaches together with adjusted rainfall indices and environmental data from this region. The results showed that both approaches (MCP and Obs) could predict the potential distribution of *S. frugiperda*. The Observation points (Obs) approach gave predicted more conservative projections compared with the Minimum Convex Polygon (MCP) approach. Areas of potential distribution that were consistently identified by the two modeling approaches included Western Afghanistan, Southern Kazakhstan and Southern Turkmenistan. The Receiver Operating Characteristic (ROC) curve test presented herein provided reliable evidence that the MaxEnt model has a high degree of accuracy in predicting the invasion of *S. frugiperda* in Central Asia.

## 1. Introduction

The fall armyworm, *Spodoptera frugiperda* (J.E. Smith) (Lepidoptera: Noctuidae), is native to tropical and subtropical regions of the Americas. It is a serious global agricultural pest insect [1] which has caused substantial maize yield losses in many countries [2]. A recent study has shown that the losses are more severe in countries with poor economies and lack of funds for pest control, such as Honduras and Argentina, where damage caused by *S. frugiperda* in maize production might reach 40% [3]. As strong migration ability and proliferation the potential for proliferation are the two important factors responsible for the worldwide spread of *S. frugiperda* [4]. *S. frugiperda* was first reported in January 2016 in West Africa [5] from where and it rapidly spread to 44 African countries within two years [3]. It has subsequently been introduced into the Indian state of Karnataka in India in July 2018 [6] and recently caused serious damages in Myanmar, Thailand and other Asian countries [7]. In January 2019, it invaded China’s Yunnan province and quickly spread to many maize producing provinces within ten months, posing a serious threat to China’s agriculture and food production systems.

Central Asia encompasses the countries from east Caspian Sea to China at about 40° N with strong solar radiation, high temperatures and vigorous evaporation. It includes the countries of Kazakhstan, Uzbekistan, Kyrgyzstan, Tajikistan, Turkmenistan and Afghanistan. The Central Asian countries have much sunlight as tropical areas with average midsummer temperatures generally between 26 °C to 32 °C. Annual precipitation varies between regions; near Aral Sea and Turkmenistan desert averages 75–100 mm; in comparison, annual precipitation of mountain areas average 1000 mm, and can reach approximately 2000 mm in the southwest of the Ferghana Mountains. Some regions receive less rain in the mountains than in the desert (Pamir’s annual precipitation is only 60 mm). Among the Central Asian countries, Afghanistan’s annual maize crop covers about 379,100 hectares, that of Kazakhstan’s corn planting covers about 140,000 hectares, Turkmenistan about 150,000 hectares, Kyrgyzstan’s corn accounts about 80,000 hectares, Uzbekistan accounts about 60,000 hectares and Tajikistan about 20,000 hectares. The spread of *S. frugiperda* depends on favorable climatic conditions, irrigation and host plants growth and vigor. Therefore, exploring the potential distribution of *S. frugiperda* in Central Asia could establish early risk warning systems in order to prepare and control measures for its potential invasion.

Ecological Niche Modeling (ENM) seeks to characterize ecological requirements of species using environmental variables associated with occurrence data to identify where suitable environmental conditions are found [8]. ENM has been used widely in biological invasion, conservation biology, biological responses to climate change, and various aspects of ecological and evolutionary biological research [9,10,11]. ENM is usually constructed from the native distribution of a species and then projected to the possible invasion site to calculate the possibility of potential distribution. This study is based on native data for *S. frugiperda* and environmental data using two modeling approaches (MCP and Obs) to project its potential distribution in the Central Asia. The specific objectives of this study were: (i) to define areas of potential invasion distribution and assess the distribution risk to provide support for early warning systems, (ii) to explore what are the main environmental factors that affect the establishment of *S. frugiperda*, and (iii) to evaluate the effects of two different buffer models on the potential distribution of the *S. frugiperda.*

## 2. Materials and Methods

### 2.1. Occurrence Data

Occurrence records of *S. frugiperda* were obtained from GBIF databases (Global Biodiversity Information Facility, https://www.gbif.org/), CPC databases (Crop Protection Compendium, https://www.cabi.org/cpc/) and related reports. We used a total of 1314 records (native range included South and Central America: 499) that reflected *S. frugiperda*’s present-day distribution. Records lacking geographic coordinates were georeferenced in Google Maps. Sampling deviation would lead to the over-fitting and thus reducing the reliability of the niche model, so we subsampled points at a 2.5 arc grid to reduce sampling bias and evaluation to minimize possible effects of spatial autocorrelation [12].

### 2.2. Environmental Variables

The environmental variables that would characterize the ecological requirements of the *S. frugiperda* were obtained from WorldClim (http://www.worldclim.org) [13]. Variables combining extreme temperature and extreme precipitation were excluded because they affected the accuracy of the modeling process [14,15]. Then we selected six variables by considering their ecological relevance and spatial correlation (Pearson < 0.9). These six variables were Mean Diurnal Range (BIO2), Isothermality (BIO3), Max Temperature of Warmest Month (BIO5), Precipitation of Wettest Month (BIO13), Precipitation Seasonality (BIO15) and Precipitation of Driest Quarter (BIO17). Considering the irrigation conditions of Central Asia, we doubled the precipitation at a 2.5 arc grid for model calibration in this study.

### 2.3. Model Calibration

**Figure 1:**
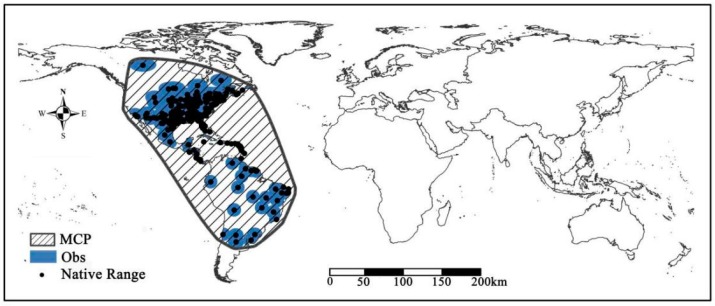
Geographic distribution of *Spodoptera frugiperda* in native range. The black dots represent the native points. MCP and Obs represent two different buffers to define the accessible areas used for niche modeling.

Among the several model algorithms were available for modeling species’ geographic distribution, MaxEnt (version 3.4.1) is the most often used popular model and follows the principle of maximum entropy [16]. In this study, we used default software conditions (convergence threshold = 10^−5^, maximum number of iterations = 500) based on two modeling approaches. The cloglog outputs with suitability values ranging from 0 (unsuitable habitat) to 1 (optimal habitat). The MaxEnt model usually calculates by selecting pseudo-absence points that exist in the model construction region for establishing the model. Therefore, the model construction region has great influence on the model prediction. According to the background selection function of model construction in SDMToolbox packages, the sample by buffered MCP and the sample by distance from obs. pts. (Obs) were used to define the model construction range (Figure 1). The predictive value presented herein traces out the high suitability value of above 0.75, and the low suitability value of below 0.25. Different colors that presented in the potential distribution map correspond to different fitting indexes.

### 2.4. Model Evaluation

Native models were fitted using the two approaches and then applied to Central Asian maps to predict the potential distributions of *S. frugiperda* in Central Asia separately. Seventy percent of native-range points were used to fit the model; the remaining 30% were used for model evaluations based on the Receiver Operating Characteristic (ROC). The Area Under Curve (AUC) is a commonly used and effective method to analyze the niche model performance [17,18]. In this study, we used the ROC curve combined with 10 times of cross validation to test the prediction results [19]. The principle of cross-validation is that it can regroup the original data into two sets (training set and test set). Hence, it not only can reduce over-fitting, but can also produce more effective information from limited data. We could then obtain a response curve with a standard deviation error bar. The AUC value was used to evaluate the model performance at a value between 0 and 1; when the AUC value was higher than 0.8, the current model was found to be superior to the random model [20].

## 3. Results

### 3.1. Model Predictions

**Figure 2:**
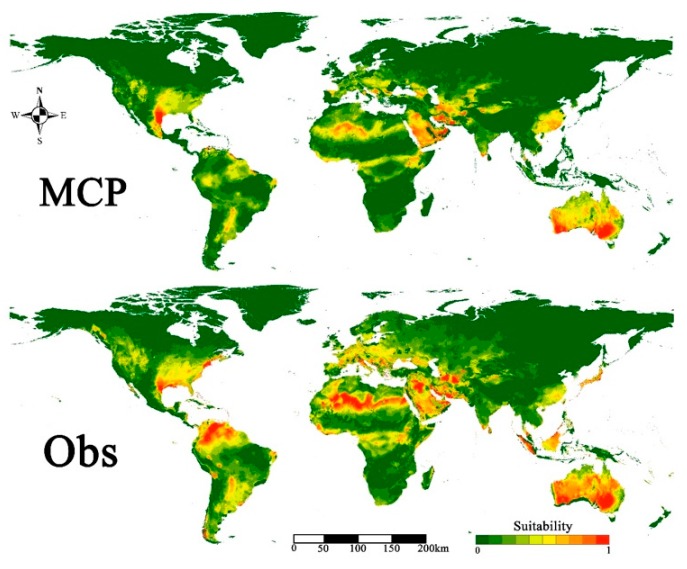
Potential habitat distribution of *Spodoptera frugiperda* in the world.

**Figure 3:**
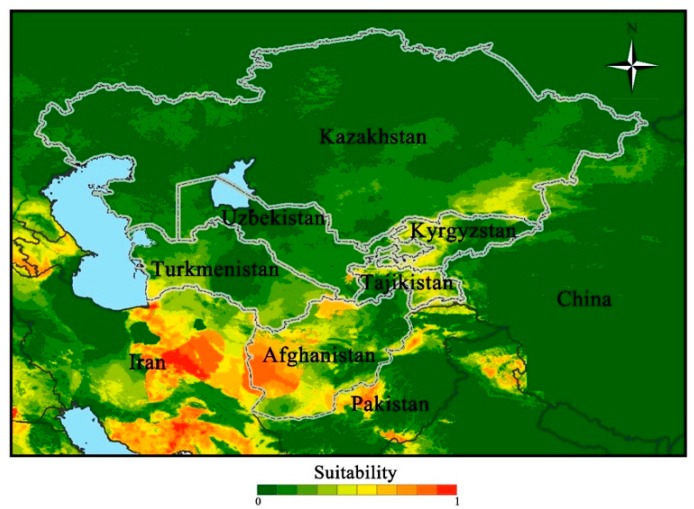
Predicted potential distribution of *Spodoptera frugiperda* in Central Asia based on the mean model.

**Figure 4:**
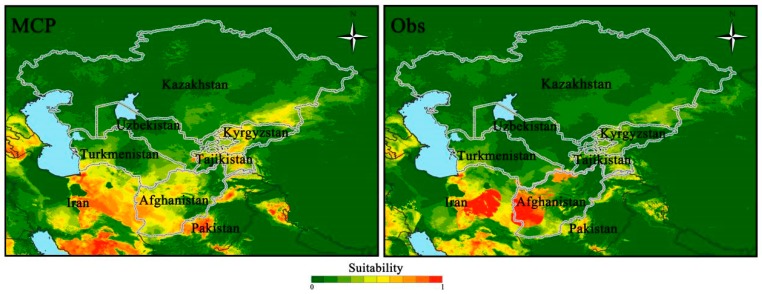
Predicted potential distribution of *Spodoptera frugiperda* in the Central Asia MCP and Obs models.

The areas of potential distributions were identified from the two models of world distribution from the Americas, Europe, Central Africa, Southern Asia and Central Australia (Figure 2). The results showed that both models could well predict the potential distribution of *S. frugiperda* in Central Asia (Figure 3). The Obs approach gave conservative predictions for the invasion of Central Asia compared to the MCP approach. Areas of potential high suitability distribution that were consistently identified by the two modeling approaches were Western Afghanistan, Southern Kazakhstan and Southern Turkmenistan (Figure 3). Predictions of mean model showed that the suitable areas were similarly concentrated in Southeast Kazakhstan, Western Kyrgyzstan and Central Tajikistan (Figure 3). The irrigated agricultural areas of Central Asia are mainly distributed in the two countries of Kyrgyzstan and Tajikistan. Predicted results obtained from adjusted precipitation values also showed that the potential distribution area of the *S. frugiperda* was located in Western Kyrgyzstan and Central Tajikistan was consistent with the actual distribution. Abundant surface irrigation water from the upper reaches of the Syr River and Amu River provide convenient conditions for local maize cultivation. Maize grown in three countries of Uzbekistan, Kazakhstan and Turkmenistan, is mostly distributed in the form of island-shaped, block-like or strip-shaped along with irrigation belts, which is located in arid and desert areas where the agricultural and animal husbandry production is more developed and the population is concentrated (Figure 4).

### 3.2. Effects of Environmental Variables

**Figure 5:**
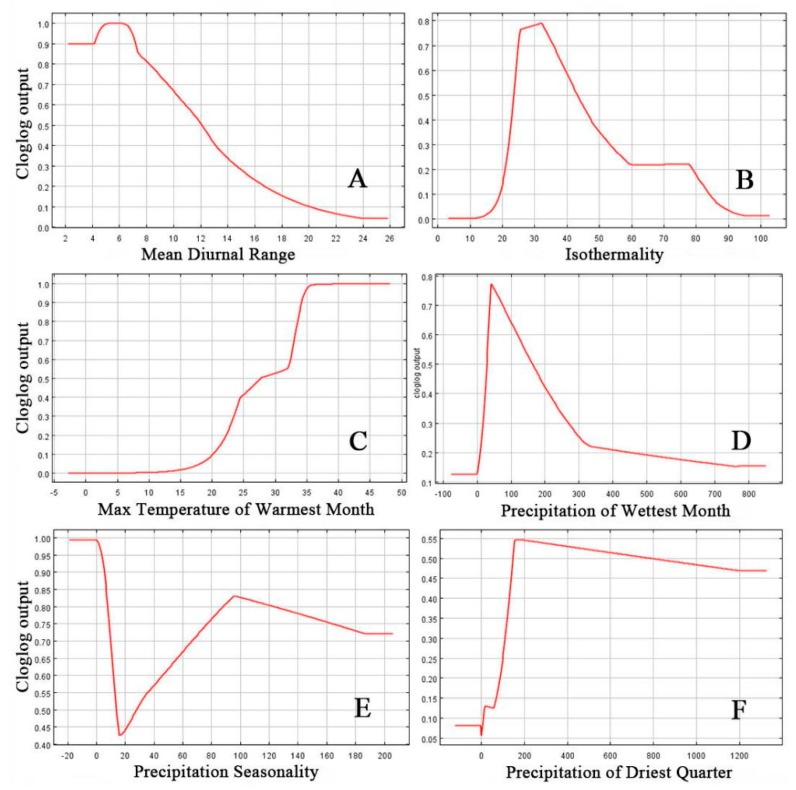
Variable response curve of Mean Diurnal Range (**A**), Isothermality (**B**), Maximum Temperature of the Warmest Month (**C**), Precipitation of the Wettest Month (**D**), Precipitation Seasonality (**E**) and Precipitation of Driest Quarter (**F**).

This study tested the effect of various environmental variables by considering their ecological relevance in predicting the potential distribution of *S. frugiperda*. Response curves of the environment variables are shown in Figure 5. In terms of temperature factors, the suitability for *S. frugiperda* was decreased with the Mean Diurnal Range (Figure 5A). The suitability of the *S. frugiperda* also has been decreased when the value of Isothermality value reached 40 °C (Figure 5B). When the Max Temperature of Warmest Month was lower than 35 °C, the suitability of *S. frugiperda* increased with increasing temperature and stabilized when the temperature exceeds 30 °C (Figure 5C). The suitability probability of *S. frugiperda* increased linearly with the increase in precipitation where the Precipitation of Wettest Month was less than 100 mm (Figure 5D); however, the suitability of *S. frugiperda* decreased when the precipitation increases in area where the Precipitation of Wettest Month was greater than 100 mm (Figure 5D). The suitability of *S. frugiperda* fell below 43% in areas where the Precipitation Seasonality was lower than 15 and reached at 83% in areas where this value was between 15 and 95; however, the suitability of *S. frugiperda* decreased gradually after the value exceeded 95 (Figure 5E). The maximum degree of suitability was 50% in some areas with the Precipitation of Driest Quarter below 150 mm; it then decreased slowly in areas where the Precipitation of Driest Quarter was greater than 150 mm (Figure 5F). Overall, the results showed that the maximum suitability of *S. frugiperda* occurred when the Diurnal Range was 6 °C, the Isothermality was 33, the Max Temperature of Warmest Month was 35 °C, the Precipitation of Wettest Month was 45 mm, the Precipitation Seasonality was 0, and the Precipitation of Driest Quarter was 180 mm.

### 3.3. Model Performance

**Figure 6:**
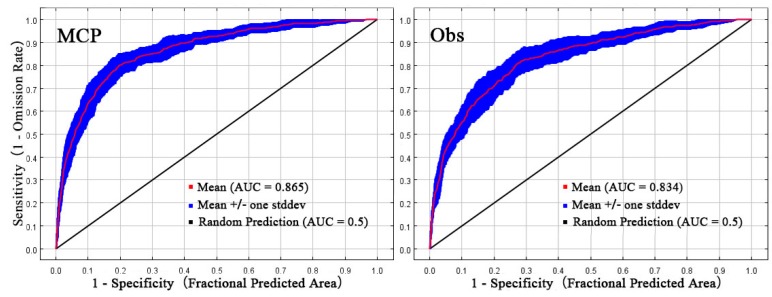
ROC curve verification of the predicted potential habitat for *Spodoptera frugiperda* by MaxEnt.

Cross-validated ROC curve results showed some differences in the MCP and Obs approaches to potential distribution of *S. frugiperda* (Figure 6). The AUC values obtained by the two models were significantly larger than their respective random prediction models (AUC = 0.5). This suggests that two predictions of the potential distribution of the *S. frugiperda* in Central Asia are reliable and the results were not random. The MCP model with a higher AUC value showed a better model performance than Obs model (MCP: AUC = 0.865, Obs: AUC = 0.834).

## 4. Discussion

The study provides a reliable basis for risk analysis combined with the ecological demand and potential distribution of the species [21]. The MaxEnt model adopted mechanical algorithms by using species’ existing and non-existing distribution points to simulate the response relationship between species and environmental factors. The MaxEnt model could avoid commission errors in predicting species distribution [11]. It showed a better predictive performance than other niche models especially when the sample points are limited [10,22]. There are differences between the models constructed for two geographical regions (MCP and Obs): The MCP approach uses the minimum polygon of species distribution point to define a minimum convex polygon for model construction, while the Obs approach mainly defines the selection of background points by buffering each species distribution point over a certain distance. The mean model approach with weighted average could improve the accuracy of prediction. Indeed, TSS combines the characteristics of sensitivity and specificity: TSS = (sensitivity + specificity − 1). However, it is susceptible to the influence of threshold, this means that different thresholds will get different results. In this study, at the threshold value of 0.5, the TSS in MPC model and Obs model projected 0.8 and 0.6, respectively. The value of 0.5 is often mentioned in the literatures. Model evaluation using AUC can be misleading although it is considered independent of threshold(s) based on the AUC combined sensitivity and specificity. The shape of ROC curve was more important than AUC value. In this study, the ROC curves of the two models show similar trends and their AUC values are not significantly different. Hence, we believe that AUC could be a sensible method to be used in assessing the accuracy of ENM.

It is apparent that the environmental dataset plays an important role in model construction, and may have a profound impact on its transferability [23]. Temperature and humidity are the main environmental factors that affect the growth, development and distribution of insects. Also, insects’ reproduction is greatly affected by the external temperature, so that reproduction is reduced whenever the temperature is too high or too low. A study by Feldmann et al. found that there is no diapause phenomenon in *S. frugiperda*, so its generation time is closely related to the external temperature [3]. Low temperature tends to decrease the birth rate and increase the death rate of the *S. frugiperda* and the adult lifespan depends on external temperature and food. Within a certain temperature range, the reproductive capacity of the female moth increases with temperatures increase [24]. However, environmental factors such as variations in higher and lower ranges of temperature, relative humidity and precipitation could be used to infer the potential distribution of species [25]. For example, the high mountains of Southeast and Central Asia block the warm and humid air from the Indian Ocean and the Pacific Oceans, thus forming a typical continental climatic regions are mountainous with a general lack of rain, extremely dry climate, and average annual precipitation below 300 mm below, and mostly in the mountains. All the six countries in Central Asia are water-deficient, which restrict planting. Under the condition that irrigation is the major need for maize production in Central Asian countries, we sought to map the potential spread of *S. frugiperda* adjusting the rainfall index. The response curve is an important result of the model output and indicates the relationship between species and environment. Moreover, it provides a check on the model results [26]. Jackknife test of variable importance generated using MaxEnt model is appropriate to examine the importance of a single environment variable in prediction process [14].

Generally speaking, the distribution of *S. frugiperda* is mostly influenced by the distribution of its host plants such as maize. The climate conditions in the major maize producing areas are mostly suitable for the occurrence and reproduction of *S. frugiperda.* Among the Central Asian countries, Afghanistan has the largest agricultural acreage and thus vulnerable to pests. Kazakhstan’s agriculture is mainly distributed in the central and southern regions with a good rainfall condition. Especially in the Southern Almaty and Chimkent, where maize is the staple crop, are high risk areas for *S. frugiperda.* The two largest states of Turkmenistan, Barkan and Ahar, are the major grain producing areas in the south. These are also the high-risk areas for the predicted results shown for the *S. frugiperda* in this study. Gizak State in eastern Uzbekistan is a major area for agricultural production and provides a suitable environment for the *S. frugiperda*. To prevent further introductions and expansions, agricultural imports and management efforts should be focused on increased genetic diversity and suitable ecological areas.

*Spodoptera frugiperda* pose a serious threat to maize production due to its invasion and feeding habits from low to high latitudes during the spring and summer and returning in the fall. The overwintering areas where pupae can survive provide an appropriate environmental conditions to ensure its successful development and reproduction. The species can overwinter from the northern Kazakhstan to the southern Turkmenistan in Central Asia, ranging from (latitudes 57° N to 35° N, which showing the transition from the Frigid-Temperate Zone to the Sub-Tropical Zone. A recent study has shown that development starts at 12.2 °C [27]. When the temperatures start to rise, *S. frugiperda* will again develop, produce larvae and invade new areas (20−30 °C).

## 5. Conclusions

*Spodoptera frugiperda* is a serious pest insect of maize and other commercial crops in Central Asia. The species tends to migrate over the winter and find areas with higher temperatures where it may develop and harm economical crops. This study confirms that both approaches tested (MCP and Obs) predict the potential distribution of *S. frugiperda*. The Observation points approach (OBs) gave more conservative projections for its Central Asia compared to the Minimum Convex Polygon (MCP) approach. Areas of potential distribution that were consistently identified by the two modeling approaches included Western Afghanistan, Southern Kazakhstan and Southern Turkmenistan. The ROC (Receiver Operating Characteristic) curve test presented herein provided reliable evidence that the MaxEnt model has a high degree of accuracy in predicting invasion of *S. frugiperda* in Central Asia. The potential distribution of the *S. frugiperda* shown in the niche models does not fully represent its current distribution due to the impact on local crops cultivation. In addition, the development and reproduction of the *S. frugiperda* is not only related to the external temperature and humidity, but also affected by light, nutrition and other factors. For example, human activities and the lack of effective biological control agents are also likely to facilitate their further invasion into novel ecologies. To prevent further pest invasions, regular pest monitoring and surveillance in the neighboring countries of Central Asia is essential.
